# Pentobarbital-Induced Myocardial Stunning in Status Epilepticus Requiring Extracorporeal Membrane Oxygenation: A Case Report and Literature Review

**DOI:** 10.1155/2016/1765165

**Published:** 2016-07-26

**Authors:** Tapan Kavi, Donna Molaie, Michael Nurok, Axel Rosengart, Shouri Lahiri

**Affiliations:** Cedars-Sinai Medical Center, 127 South San Vicente Boulevard, Suite A6600, Los Angeles, CA 90048, USA

## Abstract

*Introduction*. Mild hypotension is a well-recognized complication of intravenous pentobarbital; however fulminant cardiopulmonary failure has not been previously reported.* Case Report*. A 28-year-old woman developed pentobarbital-induced cardiopulmonary failure that was successfully treated with maximal medical management including arteriovenous extracorporeal membrane oxygenation. She made an excellent cardiopulmonary and neurological recovery.* Discussion and Conclusion*. Pentobarbital is underrecognized as a potential cause of myocardial stunning. The mechanism involves direct myocardial depression and inhibition of autonomic neuroanatomical structures including the medulla and hypothalamus. Early recognition and implementation of aggressive cardiopulmonary support are essential to optimize the likelihood of a favorable outcome.

## 1. Introduction

Continuous intravenous pentobarbital infusion is recommended and widely used for the management of refractory status epilepticus [1]. Although mild hypotension is commonly encountered, fulminant cardiopulmonary failure from pentobarbital infusion has not been reported. We report the case of a patient with status epilepticus who developed severe cardiopulmonary failure while receiving a pentobarbital infusion. She underwent extracorporeal membrane oxygenation (ECMO) and made an excellent neurological and cardiopulmonary recovery.

## 2. Case Report

### 2.1. Initial Presentation and Management

The patient is a 28-year-old woman with intractable epilepsy and no preexisting cardiac disease who presented with convulsive status epilepticus in the setting of gastroenteritis and medication intolerance. She continued to have seizures despite first-line treatment with benzodiazepines and conventional antiepileptic medications including phenytoin and levetiracetam. She was subsequently intubated and received continuous infusion midazolam and propofol drips. Neuroimaging and cerebrospinal fluid studies were unremarkable. Continuous video electroencephalography monitoring was initiated.

One day following admission to the hospital and despite high doses of continuous infusion of midazolam and propofol, electroencephalography showed sharply contoured bursts originating from right, central, and temporal leads. These bursts evolved into lateralized periodic discharges and then subsequent electrographic seizures. Lacosamide and lamotrogine failed to resolve these electrographic patterns and pentobarbital was initiated and titrated to burst suppression, which was achieved at a dose of 3 mg/kg/hour. Burst suppression was maintained for 24–48 hours after which the pentobarbital infusion rate was gradually decreased.

### 2.2. Decompensation Requiring ECMO

**Figure 1:**
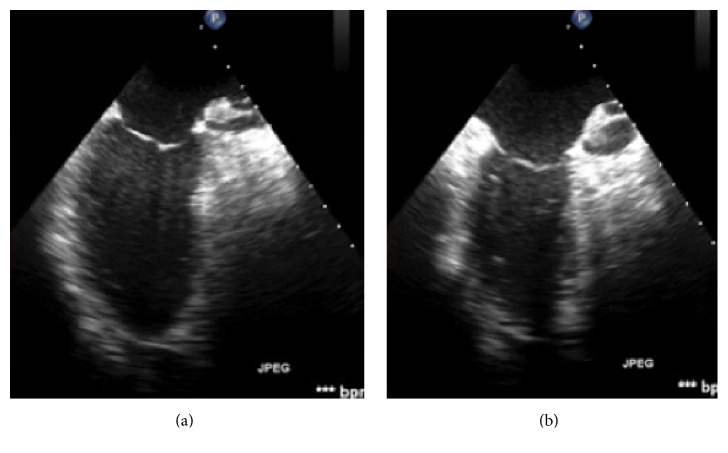
Transesophageal echocardiogram while on pentobarbital infusion, left ventricle ejection fraction of 20%: (a) end diastole; (b) end systole.

**Figure 2:**
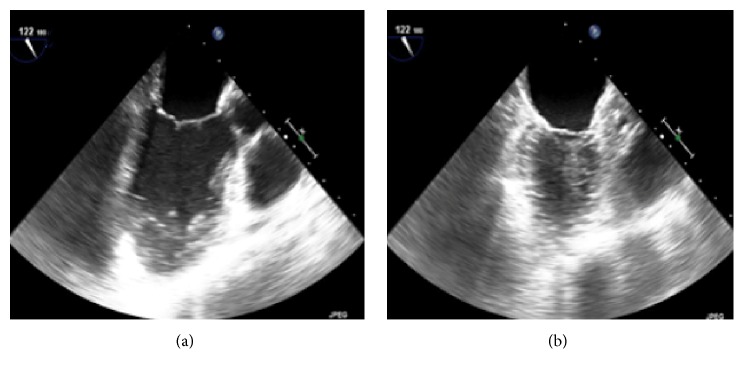
Transesophageal echocardiogram while off pentobarbital infusion, left ventricle ejection fraction of 50%: (a) end diastole; (b) end systole.

On day 11 of admission, she developed sudden onset of severe circulatory shock and hypoxia requiring four vasopressors at near maximum doses. The patient was afebrile and no infections were identified. Lactic acid level was slightly elevated at 2.5 mmol/L. By this time, the pentobarbital infusion had been tapered to 2 mg/kg/hour and the electroencephalogram (EEG) was significant for a persistent burst suppression pattern. An emergent echocardiogram was obtained and showed diminished ejection fraction of 20% (Figure 1) and hypokinesis of the basal to mid anteroseptal as well as the entire inferolateral wall. Arteriovenous ECMO was initiated and resulted in stabilization of cardiopulmonary parameters. Over the next three days, pentobarbital was gradually discontinued and electrographic ictal patterns were suppressed with midazolam. After four days on ECMO, the cardiac function improved to an ejection fraction of 50% (Figure 2). Given this rapid improvement in cardiac function, further diagnostic evaluations, such as myocardial biopsy, were not pursued. She was decannulated from ECMO and did not require further circulatory support with vasopressors.

### 2.3. Post-ECMO Management

The patient continued to have both electrographic and clinical seizures. Ketamine infusion was initiated in addition to the midazolam infusion to suppress seizures and additional conventional antiepileptic medications were progressively introduced. These included topiramate, lamotrigine, phenobarbital, perampanel, phenytoin, lacosamide, clobazam, diazepam, and pyridoxine. Further workup including magnetic resonance imaging of the brain, repeat cerebrospinal fluid analysis, and serological and urinary studies was undertaken to exclude a broad spectrum of diagnostic possibilities including porphyria, paraneoplastic encephalitides, and autoimmune encephalitides. Nevertheless, she received an empiric course of intravenous immunoglobulins and corticosteroids.

The patient underwent tracheostomy and percutaneous endoscopic gastrostomy placement on day 24. Nerve conduction study and EMG were consistent with critical illness neuromyopathy. On day 57, the patient started to keep track with her eyes, follow commands, and mouth words and began to communicate verbally. As part of the Neuroscience Intensive Care Unit initiative to improve patient and family engagement, a home visit by a neurointensivist was arranged to facilitate a video call between the patient and her mother who was bedbound at home.

## 3. Discussion

To our knowledge, this case represents the first report of pentobarbital-induced myocardial stunning and cardiopulmonary failure. Postulated biological mechanisms for cardiovascular compromise are largely derived from animal models. In addition to enhanced gamma-aminobutyric acid receptor-coupled responses and direct depression of neuronal excitability, pentobarbital's mechanism of effect in status epilepticus occurs via antagonism of brain sodium channels with inhibition of neuronal action potential propagation [2, 3]. A similar effect is seen systemically in cardiac myocytes and results in myocardial depression and stunning [3]. Cardiac index decreases to 30% within minutes of intravenous injection of pentobarbital [4]. In addition to direct myocardial injury, drug-inhibitory effects on central autonomic structures including the hypothalamus and medulla cause dysregulation of autonomic function and hemodynamic instability [5]. These inhibitory effects on the autonomic nervous system explain the extracardiac manifestations of cardiovascular compromise that have been reported in association with pentobarbital infusion. In a study on humans, Traeger et al. reported reductions in cardiac output, mean arterial pressure, and stroke volume within 12 hours of pentobarbital infusion. These abnormalities were corrected by volume resuscitation, suggesting that the hemodynamic abnormalities were related to increased venous capacitance and diminished barostatic reflex rather than direct myocardial suppression [6].

Neurogenic myocardial stunning is a well-known reversible cause of hemodynamic instability that occurs commonly in brain-injured patients due to autonomic dysregulation and catecholamine mediated myocardial injury [7]. This case report and our review of the literature suggest that pentobarbital may similarly induce reversible myocardial stunning due to inhibition of central autonomic processes as well as direct myocardial suppression. The pathophysiological course mirrored that of neurogenic myocardial stunning with full resolution of cardiac function with supportive care within days of the initial injury. Further studies are needed to examine the role of surveillance echocardiography in patients treated with pentobarbital infusions for prolonged periods of time.

Septic cardiomyopathy was considered a potential cause of cardiopulmonary deterioration due to a higher risk of infection with pentobarbital; however no active infections were diagnosed. Although status epilepticus itself may cause neurogenic myocardial stunning, continuous EEG immediately prior to and during the episode of cardiopulmonary deterioration revealed no evidence of cortical epileptic discharges.

## 4. Conclusion

This case report suggests that pentobarbital infusion may cause severe myocardial suppression and cardiopulmonary failure. A review of the literature suggests that the mechanism is complex and involves drug-inhibitory effects on the autonomic nervous system as well as direct myocardial suppression. Aggressive cardiopulmonary support is justified to optimize the likelihood of a favorable outcome.
